# Two previously undescribed triterpenoid saponins from the roots and rhizomes of *Caulophyllum robustum* Maxim

**DOI:** 10.3389/fchem.2024.1507891

**Published:** 2025-01-09

**Authors:** Cong-Yu Zhang, Yu-Mei Wang, Peng-Cheng Qiu, Jia-Yu Feng, Bing-Wen Wang, Yu Cao, Yi-Sha Wei, Yi-Tong Zhou, Hai-Feng Tang, Yun-Yang Lu, Qian Zhang

**Affiliations:** ^1^ Department of Chinese Materia Medica and Natural Medicines, School of Pharmacy, The Air Force Medical University, Xi’an, China; ^2^ School of Pharmacy, Shaanxi University of Chinese Medicine, Xianyang, China; ^3^ School of Public Health, Health Science Center, Xi’an Jiaotong University, Xi’an, Shaanxi, China

**Keywords:** Caulophyllum robustum Maxim, Triterpenoid saponin, structure identification, antitumor activity, molecular docking

## Abstract

Since ancient times, plants have provided humans with important bioactive compounds for the treatment of various diseases. Nine compounds were isolated from the roots and rhizomes of Caulophyllum robustum (a plant in the family Panaxaceae), including two new saponins C. Spanion A and C. Spanion B (1-2) and seven known saponins (3-9). The cytotoxicity of these compounds on human cancer cell lines was analyzed using MTT method. Compounds 6 and 9 exhibit cytotoxicity towards these three types of human cancer cells (<10 μM). By utilizing the SEA platform for target prediction, a common tumor related target CD81 was identified. The molecular docking of saponins 1, 2, 6, and 9 with CD81 protein showed strong binding affinities ranging from -4.5 to -7.1 kcal/mol. Research has shown that these compounds can become potential anti-tumor drugs. Further research is still recommended to understand its exact molecular mechanism and toxicological effects.

## 1 Introduction

Cancer is a widespread and highly destructive disease, and it is one of the leading causes of death in today’s society. The causes of cancer are diverse, and early symptoms are often difficult to detect, making the treatment process challenging (Siegel et al., 2021; Siegel et al., 2023). According to GLOBOCAN 2020 statistics, there are 19.29 million new cancer cases and 9.95 million deaths worldwide each year (Ferlay et al., 2021). Common chemotherapy drugs may lead to drug resistance in cancer cells due to long-term use and other reasons (Lopez and Banerji, 2017). Therefore, the search for new anti-cancer lead compounds has become particularly urgent.

From 1981 to 2019, approximately 23.5% of approved new drugs were derived from secondary metabolites of natural products (Newman and Cragg, 2020). Plants produce a wide variety of chemical substances, known as secondary metabolites. Among these secondary metabolites, saponins are a very important class of active compounds (Tian et al., 2021; Qu et al., 2023; He et al., 2023; Tian et al., 2023; Yin et al., 2023; Tian et al., 2024). Although a large number of saponin compounds have been isolated from plants, there are still some saponin components in plants that have not been isolated and studied. Therefore, further research is needed on saponins in plants to discover more compounds with anticancer activity.

Within the Berberidaceae family, *Caulophyllum* constitutes a small genus of perennial herbaceous plants. This genus comprises merely three species. *Caulophyllum robustum* is indigenous to Northeast Asia, encompassing countries such as China, Korea, and Japan. *Caulophyllum robustum* Maxim is predominantly found in regions of China like Heilongjiang, Shaanxi, and Hubei. The roots and rhizomes are known in Chinese as *Hong Mao Qi*. Extracts from it are widely employed as folk medicine in China for treating stomach aches, inflammations, irregular menstrual periods, and tumors. Modern research has shown that it has anti-inflammatory, antioxidant, anti myocardial ischemia, anti-tumor, and anti acetylcholinesterase effects (Madgula et al., 2009; Lee et al., 2012; Hideji et al., 1991; Si et al., 2010; Wang et al., 2011). Previous phytochemical studies have uncovered a series of triterpene saponins, alkaloids, and sterols in *C. robustum* Maxim. Some of these compounds possess cytotoxic properties. This indicates that there might be differences in chemical compositions of *C. robustum* Maxim grown in different areas. Hence, *C. robustum* Maxim from Mount Taibai in Shaanxi was chosen for the separation of chemical components.

**FIGURE 1:**
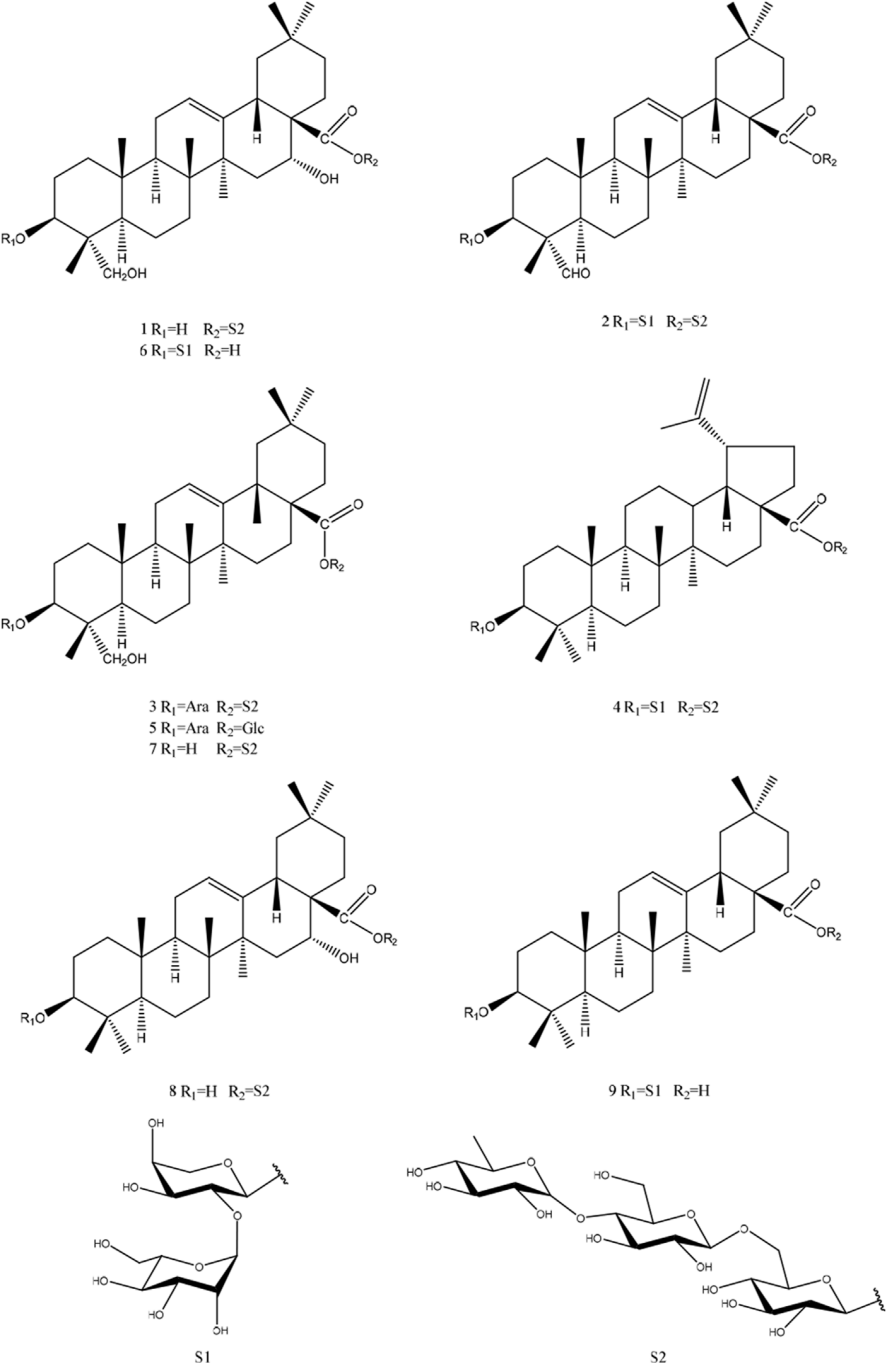
Chemical structures of compounds **1–9**.

In this study, nine triterpene saponins were obtained (Figure 1), two of which had not been previously reported in the literature (**1** and **2**). Additionally, the isolated triterpene saponins were evaluated for their cytotoxic activities, and the interactions of saponins **1**, **2**, **6**, and **9** with CD81 were described in detail. This research not only enriches our understanding of the chemical constituents of *C. robustum* Maxim but also provides potential leads for the development of new drugs targeting specific biological activities.

## 2 Experiment

### 2.1 Instruments and reagents

The separation process was performed using column chromatography (CC), including silica gel (300–400 mesh) purchased from Qingdao Marine Chemical Co., Ltd., reverse phase silica gel (RP-18, 40–63 μm) purchased from Merck&Co., Ltd. in New York, United States, and Sephadex LH-20 (GEHealthcare) purchased from Uppsala, Sweden. HPLC was performed on a Gilson PLC 2050 liquid chromatograph equipped with a Gilson PLC 2050 UV detector at a wavelength of 206 nm, using a Hedera ODS-2 column (250 × 20 mm, 10 μm, 10 nm) for semi preparation. The separated chemical reagents were purchased from Tianjin Fuyu Chemical Co., Ltd. in China. The standard samples of D-glucose (D-Glc), L-rhamnose (L-Rha), and L-arabinofuranose (L-Ara) were purchased from Sigma Aldrich Chemical Co. in St. Louis, Missouri, United States.Perform GC-MS analysis on the simadzu GC-MS QP 2010 instrument to obtain information on sugar chains, which select RXI-5 SIL MS column (30 m × 0.25 mm × 0.25 μm). ESI-MS and HRESI-MS were acquired utilizing an Agilent Q-TOF mass spectrometer (Agilent Technologies, Santa Clara, CA, United States). Optical rotation was performed on the INESA SGC-568 polarimeter (Yidian Physical Optics Co., Ltd., Shanghai, China). Conducted on a Bruker Ascend 800 spectrometer (Karlsruhe Bruker GmbH, Germany), TMS was used as an international standard for NMR. Additionally, the use of these advanced instruments and high-quality reagents ensures accurate and reliable results in the analysis and separation of complex chemical compounds. The combination of different chromatographic techniques and spectroscopic methods provides a comprehensive understanding of the chemical structures and properties of the substances under investigation.

### 2.2 Plant medicinal materials

In June 2016, the roots and rhizomes were harvested from Taibai Mountain in Shaanxi Province, China, and were authenticated as *C. robustum* Maxim by Dr. Hai-Feng Tang. A voucher specimen, designated as JB20160605, has been archived in the Herbarium of the Department of Chinese Medicinal Materials and Natural Drugs at the School of Pharmacy, Air Force Medical University in Xi’an, China.

### 2.3 Extraction and isolation

The roots and rhizomes of plants (5 kg) were chopped into pieces and extracted with 15 L of 70% ethanol at a reflux temperature of 85°C for 2 h, repeated three times. The extracts were combined and concentrated using a rotary evaporator to obtain a residue (413.2 g) Equal volumes of water were added to the residue to disperse it, followed by partitioning with petroleum ether and then with n-butanol successively. The n-butanol fraction (202.6 g) was subjected to column chromatography on silica gel, using a solvent system of CHCl_3_-MeOH-H_2_O with a volume ratio that varied from 100:1:0 to 6:3:0.5, resulting in the collection of seven fractions labeled as Fr.1 through Fr.7. Fr.4–2-1 (470 mg) underwent further purification through semi-preparative HPLC, utilizing a mobile phase of MeOH-H_2_O (60:40) at a flow rate of 8.0 mL/min, resulting in the isolation of compound **1** (5 mg, t_R_ = 26.5 min). Fr.6 (14.77 g) was processed with a Sephadex LH-20 column using CHCl_3_-MeOH(1:1) as the eluent, yielding two subfractions, Fr.6–1 and Fr.6–2. From Fr.6–1 (12.66 g), three subfractions (Fr.6–1-1 to Fr.6–1-3) were separated using an ODS column. Fr.6–1-3 (699.7 mg) was then subjected to semi-preparative HPLC with a MeOH-H_2_O (70:30) mobile phase at a flow rate of 8.0 mL/min, leading to the purification of compound **2** (23.3 mg, t_R_ = 23.5 min), **3** (28.3 mg, t_R_ = 40.1 min), and **4** (11.9 mg, t_R_ = 20.8 min). Fr.1 (5.11 g) was chromatographed on a Sephadex LH-20 column with MeOH as the eluent to obtain four subfractions (Fr.1–1 to Fr.1–4). Two subfractions (Fr.1–3-1 and Fr.1–3-2) were derived from Fr.1–3 (3.05 g) after passage through an ODS column. Fr.1–3-1 (50 mg) was further purified by semi-preparative HPLC with a MeOH-H_2_O (70:30) mobile phase at a flow rate of 8.0 mL/min, affording compound **5** (19.8 mg, t_R_ = 24.1 min). Compound **6** (20 mg, t_R_ = 17.8 min) was extracted from Fr.1-3-1-2 (40.02 mg) using HPLC with a MeOH-H_2_O (70:30) eluent at a flow rate of 8.0 mL/min. Fr.3 (2.73 g) was purified over a Sephadex LH-20 column with MeOH as the eluent, resulting in two subfractions (Fr.3–1 and Fr.3–2). Two subfractions (Fr.3–1-1 and Fr.3–1-2) were obtained from Fr.3–1 (0.72 g) following passage through an ODS column. Fr.3–1-2 (100 mg) was further purified by semi-preparative HPLC using a MeOH-H_2_O (70:30) mobile phase at a flow rate of 8.0 mL/min, yielding compounds **7** (55.9 mg, tR = 30.5 min) and **8** (6.8 mg, t_R_ = 43.3 min). Fr.4 (5.89 g) was processed on a Sephadex LH-20 column with MeOH as the eluent to yield two subfractions (Fr.4–1 and Fr.4–2). Five subfractions (Fr.4–2-1 to Fr.4–2-5) were separated from Fr.4–2 (4.47 g) using an ODS column. Fr.4–2-5 (60 mg) was then purified by semi-preparative HPLC with a MeOH-H_2_O (60:40) mobile phase at a flow rate of 8.0 mL/min, resulting in the isolation of compound **9** (169.8 mg, t_R_ = 20.7 min).

#### 2.3.1 Compound **1**

Amorphous solid; [α] −10.6 (*c* 0.54, MeOH); ^1^H (800 MHz, pyridine-*d5*) and ^13^C (200 MHz, pyridine-*d5*) NMR data see Table 1; HR-ESI-MS m/z 957.5076 [M - H]^-^ (calcd. For C_48_H_78_O_19_, 957.5059).

**TABLE 1:** ^13^C NMR and ^1^H NMR data of **1** in pyridine-*d*_*5*_.

Position (aglycone)	*δ*_C_	*δ*_H_	Position (sugar)	*δ*_C_	*δ*_H_
1a	39.0	1.14 m	28-O-Glc Ⅰ-1′	95.9	6.26, d (8.5)
1b		1.65 m	2’	74.0	4.08 m
2a	27.8	1.97 m	3’	78.8	4.21, m
2b		2.20 m	4’	70.9	4.29 m
3	73.7	4.22 m	5’	78.1	4.11 m
4	42.9		6’a	69.3	4.33 m
5	48.8	1.57 m	6’b		4.68 m
6a	18.7	1.64 m	Glc Ⅱ-1’’	105.0	4.99, d (8.0)
6b		1.53 m	2’’	75.4	3.95 m
7a	33.3	1.44 m	3’’	76.5	4.17 m
7b		1.78 m	4’’	78.3	4.44 m
8	40.2		5’’	77.2	3.68 m
9	47.4	1.95 m	6’’a	61.4	4.11 m
10	37.4		6’’b		4.22 m
11	24.0	2.06 m	Rha-1’’’	102.8	5.88 s
12	122.9	5.64, br s	2’’’	72.7	4.70 m
13	144.5		3’’’	72.8	4.58 m
14	42.2		4’’’	74.1	4.36 m
15a	36.3	1.77, o	5’’’	70.4	4.99 m
15b		2.57, br d (12.2)	6’’’	18.6	1.73, d (6.5)
16	74.4	4.29, br s			
17	49.2				
18	41.3	3.54, dd (4.0, 4.0)			
19a	47.3	1.37 m			
19b		2.79, t (12.2)			
20	30.9				
21a	36.0	1.30 m			
21b		2.42 m			
22a	32.3	2.45 m			
22b		2.18 m			
23a	68.1	4.15 m			
23b	68.1	3.69 m			
24	13.2	1.09 s			
25	16.3	1.09 s			
26	17.8	1.23 s			
27	27.3	1.80 s			
28	176.1				
29	33.2	0.98 s			
30	24.7	1.05 s			

#### 2.3.2 Compound **2**

Amorphous solid; [α] +9.6 (*c* 0.24, MeOH); ^1^H (800 MHz, pyridine-*d5*) and ^13^C (200 MHz, pyridine-*d5*) NMR data see Table 2; HR-ESI-MS m/z 1233.5892 [M - H]^-^ (calcd. For C_59_H_94_O_27_, 1233.5904).

**TABLE 2:** ^13^C NMR and ^1^H NMR data of **2** in pyridine-*d*_5_.

Position (aglycone)	*δ*_C_	*δ*_H_	Position (sugar)	*δ*_C_	*δ*_H_
1a	39.0	0.92 m	3-O-Ara-1’	103.8	4.84, d (6.0)
1b		1.53 m	2’	81.8	4.48 m
2a	25.8	1.88 m	3’	74.7	4.30 m
2b		2.09 m	4’	69.2	4.34 m
3	83.2	3.98 m	5’a	66.2	4.29 m
4	56.4		5’b		3.75 m
5	48.8	1.32 m	Glc Ⅰ-1’’	106.6	5.13, d (7.5)
6a	21.3	0.92 m	2’’	77.1	4.09 m
6b		1.38 m	3’’	79.5	4.21 m
7a	33.2	1.76 m	4’’	71.7	4.33 m
7b		1.87 m	5’’	79.1	4.42 m
8	41.0		6’’a	63.4	4.44 m
9	48.8	1.67 m	6’’b		4.53 m
10	37.0		28-O-Glc Ⅱ-1’’’	96.5	6.24, d (8.0)
11a	24.1	0.90 m	2’’’	74.5	4.13 m
11b		1.93 m	3’’’	78.9	4.10 m
12	123.4	5.41, br s	4’’’	72.1	4.29 m
13	145.8		5’’’	79.1	4.18 m
14	43.1		6’’’a	70.0	4.34 m
15a	29.0	1.10 m	6’’’b		4.68 m
15b		2.26 m	Glc Ⅲ-1’’’	105.7	4.99, d (8.0)
16a	24.6	1.90 m	2’’’	76.2	3.95 m
16b		2.06 m	3’’’’	77.4	4.15 m
17	47.2		4’’’’	79.0	3.84 m
18	42.5	3.18, dd (10.2, 3.5)	5’’’’	78.0	3.67 m
19a	47.0	1.24 m	6’’’’a	62.1	4.10 m
19b		1.75 m	6’’’’b		4.21 m
20	31.6		Rha-1’’’’’	103.6	5.87 s
21a	34.8	1.12 m	2’’’’’	73.4	4.69 m
21b		1.33 m	3’’’’’	73.6	4.56 m
22a	33.3	1.37 m	4’’’’’	74.8	4.34 m
22b		1.16 m	5’’’’’	71.2	4.96 m
23	209.5	9.97 s	6’’’’’	19.4	1.71 m
24	11.4	1.39 s			
25	16.6	0.91 s			
26	18.3	1.07 s			
27	26.9	1.22 s			
28	177.3				
29	34.0	0.91 s			
30	24.5	0.90 s			

### 2.4 Sugar analysis of new compounds

The gas chromatography-mass spectrometry (GC-MS) technique was employed to analyze the monosaccharide content of compounds **1** and **2**, adhering to a procedure akin to those reported in existing scientific literature. Each compound, weighing 5 mg, was subjected to hydrolysis by being treated with 1 mL of 2 M trifluoroacetic acid (TFA) at a temperature of 110°C for a duration of 90 min. Following hydrolysis, acetylation was performed using acetic anhydride. The reaction mixture was then diluted with 2 mL of distilled water and reduced using 100 mg of sodium borohydride (NaBH_4_). After reduction, the mixture was re-acetylated with acetic anhydride at 100°C for 60 min. Standard monosaccharide controls—D-glucose (D-Glc), L-rhamnose (L-Rha), and L-arabinose (L-Ara), each at a concentration of 5 mg—were processed using the identical method and analyzed via GC–MS.

The GC-MS analysis was conducted under the specified conditions: the column temperature was ramped from 130°C to 180°C at a rate of 3°C per minute and held at 180°C for 5 min. Subsequently, the temperature was programmed to increase to 310°C at a rate of 10°C per minute, where it was maintained for 15 min. The temperatures of both the injector and the flame ionization detector (FID) were set to 285°C. Nitrogen gas (N_2_) with a purity of at least 99.999% was used as the carrier gas, flowing at a rate of 20.0 mL per minute. The retention times for the standard monosaccharides were recorded as follows: D-Glc at 12.89 min, L-Rha at 5.65 min, and L-Ara at 5.15 min.

### 2.5 Cytotoxicity assay

The cytotoxicity of compounds **1**–**9** against human cancer cells was detected using the MTT method in 96-well microplates. The DMEM or RPMI-1640 culture medium (Hyclone, United States) was supplemented with 10% FBS (GIBCO, United States), 1% penicillin, and streptomycin (Elabscience Biotechnology Co., Ltd., Wuhan, China), in which the cell lines were cultured at 37°C with 5% CO_2_. Cells in the logarithmic phase (5000 cells/well) were seeded in 96-well plates (100 μL/well) incubated for 24 h, then treated with various concentrations of saponins **1**–**9** (<0.1% DMSO) for 24 h, separately. Then, 20 μL MTT reagent (Sigma Biotechnology Co., Ltd.) was added to each well incubated for 2 h at 37°C with 5% CO_2_. The optical density of each well was measured using a microplate reader (BioTek, United States) at a wavelength of 450 nm. The IC_50_ values of saponins **1**–**9** were evaluated according to their optical densities. The experiment was conducted three independent replicates, with doxorubicin and nimustine hydrochloride (Sigma, purity ≥99%) used as positive controls.

### 2.6 Target prediction and molecular docking

By means of the SEA platform (https://sea16.docking.org/), the target prediction of compounds with favorable activity was carried out, and common targets were selected for molecular docking simulation.

Molecular docking was employed to depict the interactions between proteins and ligands. The protein CD81, identified by its UniProt accession number P60033, was sourced from the UniProt database (https://www.uniprot.org/). Meanwhile, the ideal molecular configurations for compounds **1**–**9** were derived from Chem3D. In the molecular docking process, the structure of CD81 was modified using PyMOL 2.5.4 and AutoDock Tools 1.5.6 software. Additionally, the affinity between CD81 and its ligand was assessed using Auto Dock Vina 1.1.2 software. PyMOL 2.5.4 was utilized to visualize the molecular docking outcomes. This comprehensive approach of target prediction and molecular docking provides valuable insights into the potential mechanisms of action of these compounds. It helps in understanding how the compounds interact with specific proteins and may offer clues for further research and development of novel therapeutics.

## 3 Results

### 3.1 Isolated phytochemicals from *Caulophyllum robustum* Maxim

The Liebermann-Burchard and Molish tests indicated that compound **1** (**1**) was a saponin (Liu et al., 2023). The molecular formula was identified as C_56_H_88_O_27_ by HR-ESI-MS at m/z 957.5076 [M - H]^-^ (calcd. For C_48_H_78_O_19_, 957.5059).The NMR spectrum (Table 1) showed six methyl signals at *δ*_H_ 0.98 (s, H-29), 1.05 (s, H-30), 1.09 (s, H-24), 1.09 (s, H-25),1.23 (s, H-26),1.80(s, H-27), an ene proton *δ*_H_ 5.64 (br s, H-12). Correspondingly, six methyl carbon signals at *δ*_C_ 13.2 (C-24),16.3 (C-25),17.8 (C-26),24.7 (C-30),27.3(C-27),33.2 (C-29). Meanwhile, one oxygenated methine δ_C_ 73.7 (C-3) and δ_C_ 74.4 (C-16), two olefinic carbons at *δ*_C_ 122.9 (C-12) and 144.5 (C-13), as well as one carboxyl at *δ*_C_ 176.1 (C-28) can be found in the ^13^C NMR spectrum (Table 1). The NOESY spectrum revealed correlations between H-3/H-23 and H-3/H-5, which pointed to the β-configuration of the oxygen atom at the C-3 position. In the ^1^H–^1^H COSY spectrum, the proton signal is correlated with the two proton signals of the methylene group at *δ*_H_ 2.57 (br d, 1H, J = 12.2 Hz, H-15b) and *δ*_H_ 1.77 (15a).Based on the broad single peak of the hydrogen proton on the methylene carbon in the ^1^H-NMR, HMBC and DEPT spectra, it is inferred that the hydroxyl group at position 16 should be in the alpha configuration (Shashi and Mahato, 1994). Taken together, the spectroscopic of the aglycone closely matched that of caulophyllogenin (Strigina et al., 1974).

**FIGURE 2:**
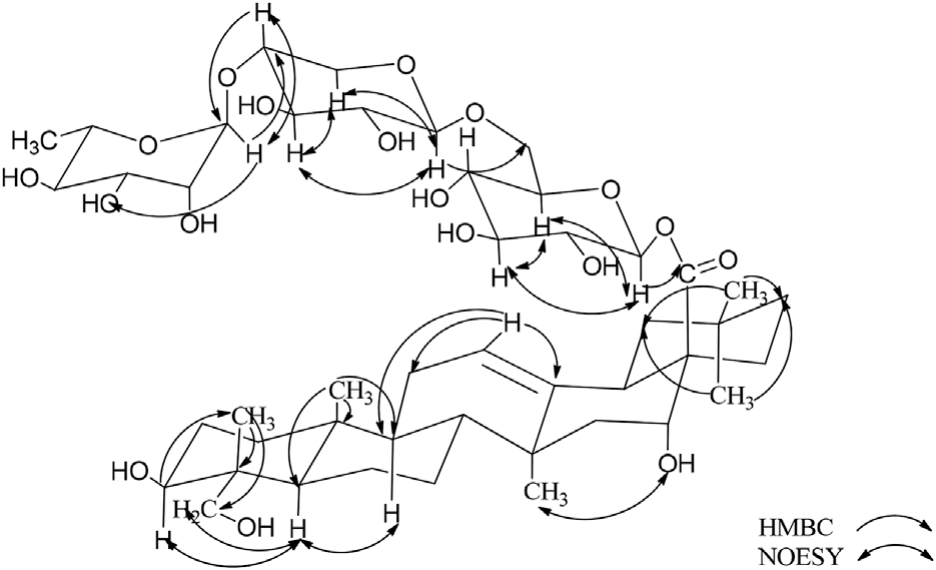
Key HMBC and NOESY correlations of compoud **1**.

According to the results of GC-MS analysis, after acid hydrolysis treatment three monosaccharides were identified as D-Glc and L-Rha, and the ratio of them is 2:1. By analyzing ^1^H NMR, the hydrocarbon signals of three sugar groups were assigned (Table 1). The ^1^H NMR spectrum of **1** exhibited three sugar anomeric protons at *δ*_H_ 6.26 (d, *J* = 8.5 Hz, Glc’ H-1), *δ*_H_ 4.99 (d, *J* = 8.0 Hz, Glc’’ H-1) and 5.88 (s, Rha’ H-1), which showed HSQC correlations with the anomeric carbon signals at *δ*_C_ 95.9, 105.0 and 102.8, respectively. The trisaccharide chain was established to be Rha (1→4)-Glc ((1→6)-Glc- by the observation of HMBC correlations from Glc-H-1′ to C-28 of the aglycone, Glc-H-1′′ (*δ*_H_ 4.99) to Glc-C-6′ (*δ*_C_ 69.3) and Rha-H-1‴(*δ*_H_ 5.87) to Glc-C-4′′ (*δ*_C_ 78.3). This conclusion was confirmed by the NOESY correlations, as shown in Figure 2. Therefore, the chemical structure of 1 was elucidated as caulophyllogenin-28-*O*-*α*-L-rhamnopyranosyl-(1→4)-*β*-D-glucopyranosyl-(1→6)-*β*-D-glucopyranosyl ester, named *C.* Spanion A.

Compound **2** (**2**) is a white, amorphous substance. When we used compound **2** (**2**) for Lieberman-Burchard and Molish tests, the results of both experiment were showed positively, indicating that compound 2 may be a saponin (Liu et al., 2023). Its molecular formula was established as C_59_H_94_O_27_ by HR-ESI-MS at m/z 1233.5892 [M - H]^-^ (calcd. For C_48_H_78_O_19_, 1233.5904). As compound **2** are similar to compond **1**, indicating that they may gain similar structures (Table 2). The C-3 carbon was observed at δ_C_ 83.2, which suggested that the sugar linkage was formed at C-3. The main difference existed in the chemical shifts of H-16 and H-23 (*δ*_H-16_ 1.90, 2.06 and *δ*_H-23_ 9.97 for **2** and *δ*_H-16_ 4.29 and *δ*_H-23_ 4.15, 3.69 for **1**). Furthermore, the aglycone’s spectroscopic details were found to be highly consistent with those of gypsogenin (Zhou et al., 1989).

**FIGURE 3:**
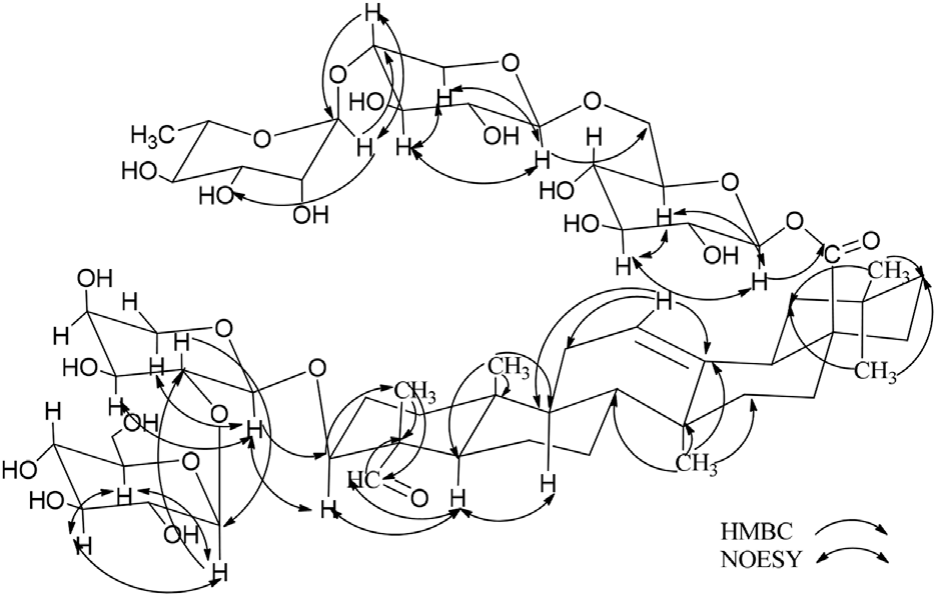
Key HMBC and NOESY correlations of compoud **2**.

By using the same method as described above, the results of GC-MS indicating the existence of D-Glc, L-Rha and L-Ara, and the ratio of them is 3:1:1. Comparing the NMR spectra and extensive 2D NMR studies, we found that 2 and 1 have the same linked trisaccharide chain Aglycone C-28. Through the analysis of HMBC spectrum of 2, Ara was connected to C-3 of the aglycone as a cross-peak exists between C-3 of the aglycone and H-1 of Glc I. Key HMBC and NOESY shows in Figure 3. Therefore, the chemical structure of **2** was elucidated as 3-O-α-L-glucopyranosyl-(1→2)-α-L-arabinopyranosyl-gypsogenin-28-O-α-L-rhamnopyranosyl-(1→4)-β-D-glucopyranosyl-(1→6)-β-D-glucopyranosyl ester, named *C.* Spanion B.

By correlating the NMR data with the information documented in the existing literature, the known saponins were characterized as 3-*O*-*α*-L-arabinopyranosyl-hederagenin-28-*O*-*α*-L-rhamnopyranosyl-(1→4)-*β*-D-glucopyranosyl-(1→6)-*β*-D-glucopyranosyl ester (**3**) (Wang et al., 2010), 3-*O*-*α*-L-glucopyranosyl-(1→2)-*α*-L-arabinopyranosyl-betulinic acid 28-*O*-*α*-L-rhamnopyranosyl-(1→4)-*β*-D-glucopyranosyl-(1→6)-*β*-D-glucopyranosyl ester (**4**) (Liu et al., 2021), HN-saponin F (**5**) (Fumie et al., 1990), cauloside C (**6**) (Anisimov and Chaikina, 2015), Kalopanax saponin G (**7**) (Miyakoshi et al., 1999), echinocystic acid-28-*O*-*α*-L-rhamnopyranosyl-(1→4)-*β*-D-glucopyranosyl-(1→6)-*β*-D-glucopyranosyl ester (**8**) (Xia et al., 2014), oleanolic acid 3-*O*-*β*-D-glucopyranosyl-(1→2)-*α*-L-arabinopyranoside (9) (Sobolev et al., 2000). See Supplementary Data Sheet 1 for relevant data.

### 3.2 Cytotoxicity assay

The MTT assay was used to determine the cytotoxic properties of compounds **1**–**9**, and the findings are outlined in Table 3. Notably, compounds **6** and **9** demonstrated potent cytotoxic effects (IC_50_ ≤ 10 μM) against a panel of three human cancer cell lines: U251MG, HepG2, and HL-60. Compound **1** exhibits weak activity against HL-60(IC_50_ = 56.45 ± 5.18 μM), while compound **2** shows weak activity against U-251MG (IC_50_ = 76.67 ± 4.25 μM).

**TABLE 3:** Cytotoxicity of compounds **1-9**.

Compounds	Cytotoxicity (IC_50_, μM; mean ± SD, n = 5)		
	HL-60	Hep-G2	U-251MG
Doxorubicin^a^	0.35 ± 0.05	0.50 ± 0.15	—
ACNU^a^	—	—	1.12 ± 0.25
1	56.45 ± 5.18	>80	>80
2	>80	>80	76.67 ± 4.25
3	>80	>80	>80
4	>80	>80	>80
5	>80	>80	>80
6	2.56 ± 1.05	4.76 ± 2.03	5.81 ± 3.25
7	>80	>80	>80
8	57.71 ± 2.38	>80	>80
9	1.58 ± 0.05	3.58 ± 0.49	4.40 ± 0.13

### 3.3 Target prediction and molecular docking

#### 3.3.1 Target prediction

Target prediction of compounds 1, 2, 6, and 9 through the SEA platform. Take the intersection of the targets of four compounds and select CD81 as the target.

#### 3.3.2 Molecular docking

**FIGURE 4:**
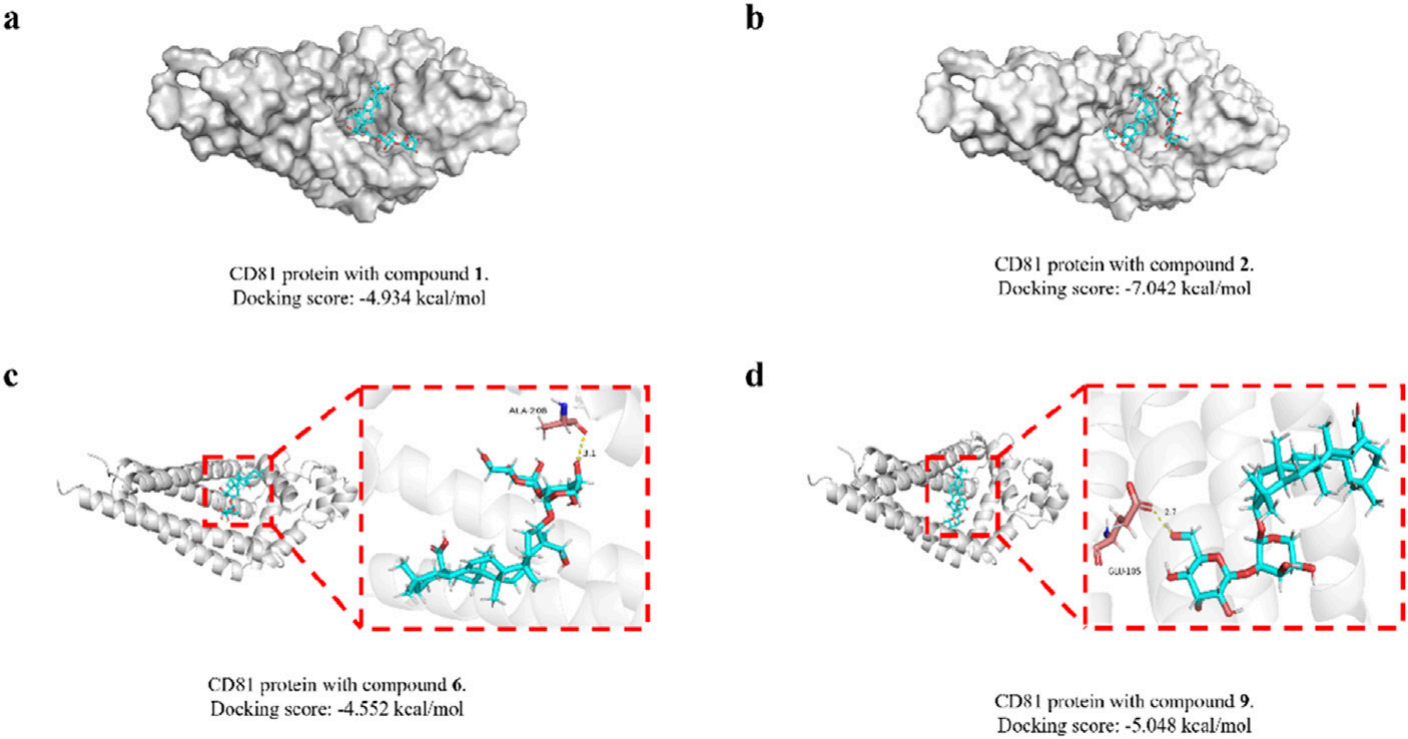
Molecular docking modeling of saponins **1**, **2**, **6** and **9** with CD81. Compound **1 (A)**; compound **2 (B)**; compound **6 (C)**; compound **9 (D)**.

We then molecularly docked the proteins and compounds to study their binding patterns and interactions. The protein is first pretreated (including hydrogenation, water removal, energy minimization, etc.), followed by the formation of a lattice with coordinates X: 21.42, Y: 36.31, Z: 21.35 and a size of 20 Å. Finally, the treated compound is docked with the protein. By docking, we found that both compounds and proteins had acceptable binding energy (the Docking score, the smaller the value, the stronger the binding energy, generally less than −5 to prove a strong binding energy). To our excitement, compound **2** was the strongest with protein, reaching −7.042 kcal/mol, proving excellent affinity between the compound and protein. In addition, we studied the binding patterns and interactions between the compounds and proteins, as shown in the figure above. Each compound can bind to an active pocket of the protein. Specifically, for compound **6**, the compound can form 1 hydrogen bond interaction with ALA-208 on the protein. For compound **9**, the compound can form a hydrogen bond with GLU-105 on the protein.The visual representations of these interactions are depicted in Figure 4.

## 4 Discussion

The study on *in vitro* anti-tumor activity found that compounds **6** and **9** have strong cytotoxicity against HL-60, Hep-G2, and U-251MG cells. Among them, compound **6** has significant activity against HL-60, Hep-G2, and U-251MG cells, with IC_50_ values of 2.56, 4.76, and 5.81 μM, respectively; compound **9** exhibits significant activity against HL-60, Hep-G2, and U-251MG cells, with IC_50_ values of 1.58, 3.58, and 4.40 μM. Regrettably, the remaining seven compounds, including two novel entities (**1** and **2**), that formed glycosides at the 28th position, exhibited diminished cytotoxicity against the aforementioned cancer cells. Nonetheless, compound **1** displayed moderate cytotoxicity towards HL-60 cells, and compound **2** showed a modest effect on U251MG cells. As illustrated in Table 3, the presence of carboxyl groups at the 28th position is associated with enhanced cytotoxicity in comparison to glycosides at the same position. Additionally, it is observed that the saponin activity of the oleanolic acid type is notably robust.

CD81 is involved in several crucial cellular activities, including the arrangement of the cell membrane, the movement of proteins, the merging of cells, and interactions between cells. Within the immune system, CD81 plays a role in managing the immune synapse, the gathering of receptors, and the transmission of signals. It also influences the suppression of both adaptive and innate immune responses. It is found in a broad spectrum of cancers, such as those affecting the breast, lungs, prostate, skin (melanoma), brain, and lymph nodes. The level of CD81 expression—whether it is increased or decreased—has been linked to different outcomes in cancer prognosis, with implications for both favorable and unfavorable outcomes (Vences-Catalán et al., 2017). All of these binding energies are below the threshold of −4.5 kcal/mol, suggesting a strong interaction. The findings suggest a possible link between the cytotoxic effects of these saponins on tumor cells and their interaction with CD81. This relationship warrants further investigation in subsequent research to elucidate the underlying mechanisms.

## 5 Conclusion

A total of nine compounds were isolated and identified, comprising two new compounds and seven known ones. The anticancer activities of these compounds were assessed using the MTT method, and it was found that compounds 1, 2, 6 and 9 have potential anticancer properties. The target prediction and molecular docking results for these compounds suggest that saponin compounds may have some correlation with the CD family of proteins. This discovery implies that saponin compounds might not only kill tumor cells through cytotoxicity but could also possess a novel mechanism for combating tumor cells. This suggests that these compounds may serve as potential anti-cancer precursor compounds for further investigation. Regrettably, we have not yet been able to validate this phenomenon, and there is a lack of experimental evidence to confirm whether saponins indeed bind to CD family proteins and how they exert their cytotoxic effects on tumor cells.

## Data Availability

The original contributions presented in the study are included in the article/Supplementary Material; further inquiries can be directed to the corresponding authors.
