# Supplementary figures and images for: Two previously undescribed triterpenoid saponins from the roots and rhizomes of Caulophyllum robustum Maxim
Source: Front Chem. 2025 Jan 9;12:1507891. doi: 10.3389/fchem.2024.1507891 (PMC11754256; doi:10.3389/fchem.2024.1507891)

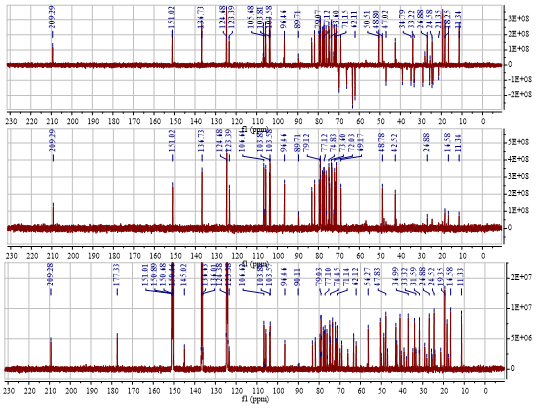

Supplement: Supplementary file 3 [file Supplementaryfile2.zip › data of 2/13C-NMR and DEPT (135°and 90° ) spectrum of saponin 2.tif]

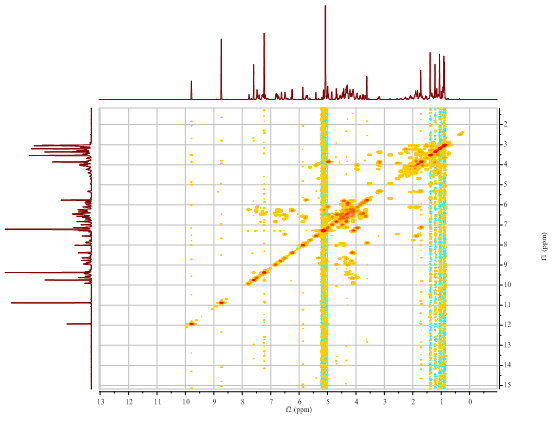

Supplement: Supplementary file 3 [file Supplementaryfile2.zip › data of 2/1H-1H COSY spectrum of saponin 2.tif]

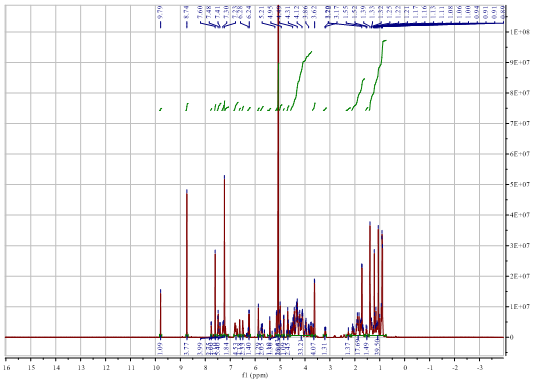

Supplement: Supplementary file 3 [file Supplementaryfile2.zip › data of 2/1H-NMR spectrum of saponin 2.tif]

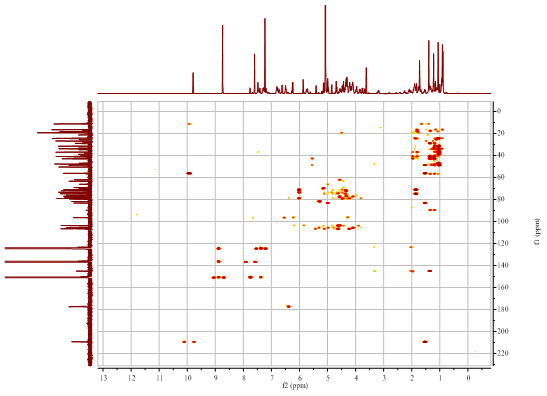

Supplement: Supplementary file 3 [file Supplementaryfile2.zip › data of 2/HMBC spectrum of saponin 2.tif]

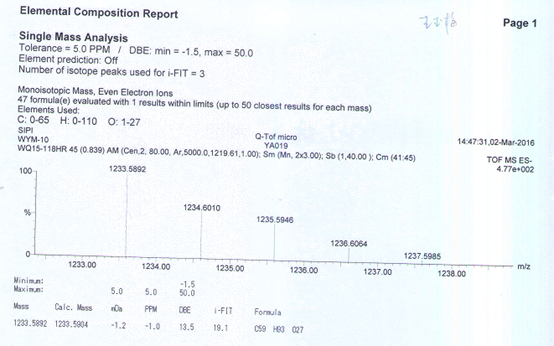

Supplement: Supplementary file 3 [file Supplementaryfile2.zip › data of 2/HR-ESI-MS of saponin 2.tif]

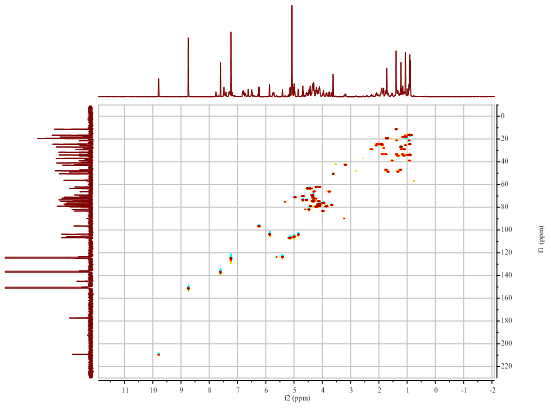

Supplement: Supplementary file 3 [file Supplementaryfile2.zip › data of 2/HSQC spectrum of saponin 2.tif]

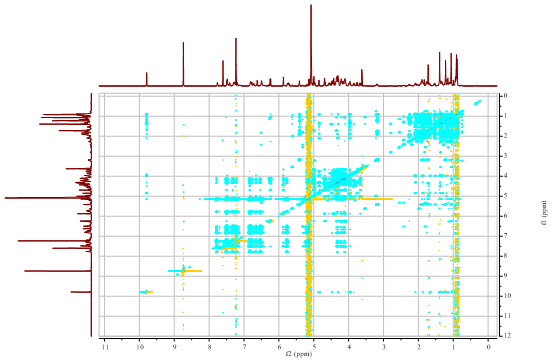

Supplement: Supplementary file 3 [file Supplementaryfile2.zip › data of 2/NOESY spectrum of saponin 2.tif]

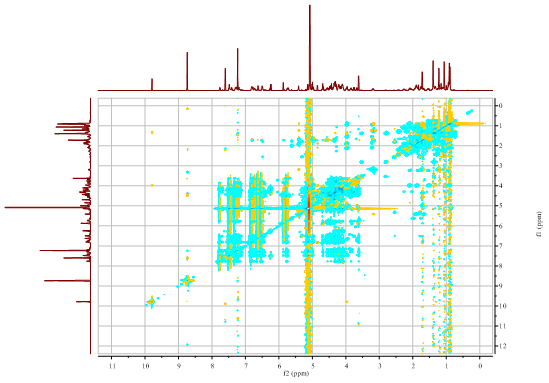

Supplement: Supplementary file 3 [file Supplementaryfile2.zip › data of 2/TOCSY spectrum of saponin 2.tif]

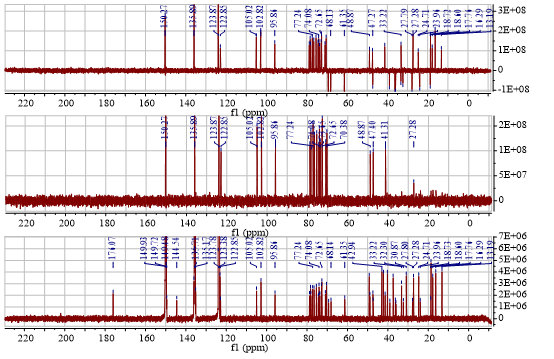

Supplement: Supplementary file 4 [file Supplementaryfile1.zip › data of 1/13C-NMR and DEPT (135°and 90° ) spectrum of saponin 1.tif]

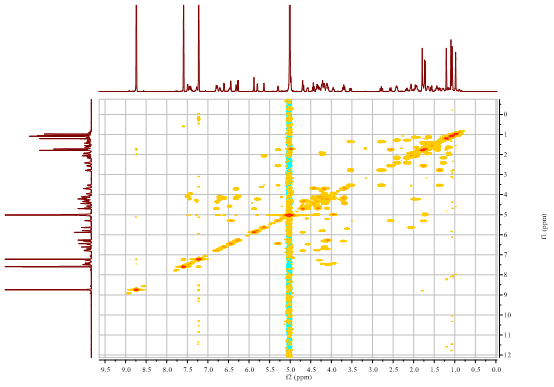

Supplement: Supplementary file 4 [file Supplementaryfile1.zip › data of 1/1H-1H COSY spectrum of saponin 1.tif]

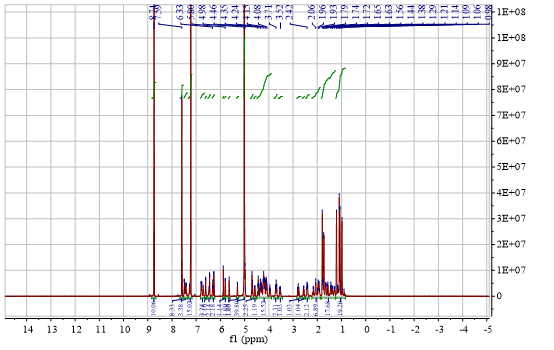

Supplement: Supplementary file 4 [file Supplementaryfile1.zip › data of 1/1H-NMR spectrum of saponin 1.tif]

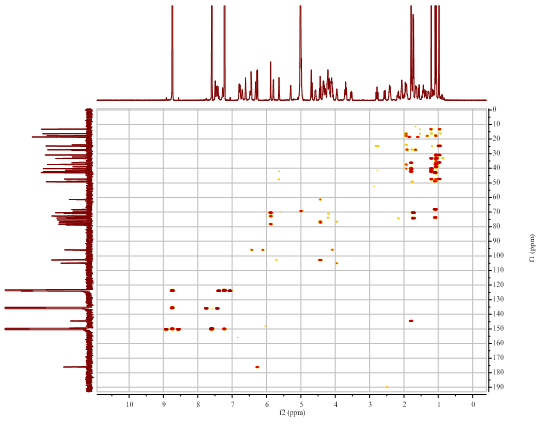

Supplement: Supplementary file 4 [file Supplementaryfile1.zip › data of 1/HMBC spectrum of saponin 1.tif]

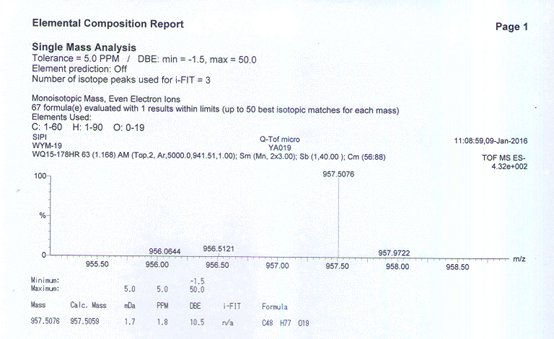

Supplement: Supplementary file 4 [file Supplementaryfile1.zip › data of 1/HR-ESI-MS of saponin 1.tif]

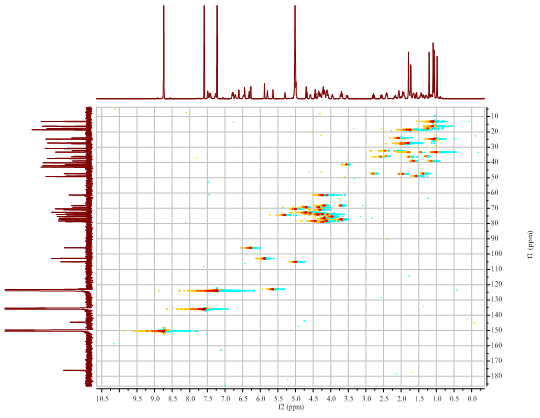

Supplement: Supplementary file 4 [file Supplementaryfile1.zip › data of 1/HSQC spectrum of saponin 1.tif]

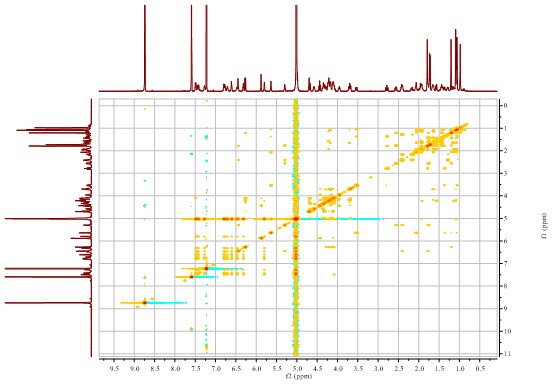

Supplement: Supplementary file 4 [file Supplementaryfile1.zip › data of 1/NOESY spectrum of saponin 1.tif]

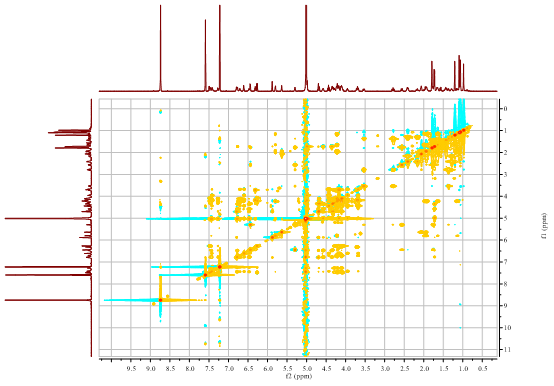

Supplement: Supplementary file 4 [file Supplementaryfile1.zip › data of 1/TOCSY spectrum of saponin 1.tif]
