# Supplementary material for: Two previously undescribed triterpenoid saponins from the roots and rhizomes of Caulophyllum robustum Maxim
Source: Front Chem. 2025 Jan 9;12:1507891. doi: 10.3389/fchem.2024.1507891 (PMC11754256; doi:10.3389/fchem.2024.1507891)
Supplement: Supplementary file 5 [file DataSheet1.docx]

**Table S1** ^13^C- NMR (125 MHz) chemical shifts saponins **3**-**6** in pyridine-*d_5_*

| C | **3** | **4** | **5** | **6** | C | **3** | **4** | **5** | **6** |
| --- | --- | --- | --- | --- | --- | --- | --- | --- | --- |
| 1 | 39.0 | 40.2 | 39.4 | 38.6 | 3-O-sugar |  |  |  |  |
| 2 | 26.2 | 27.3 | 26.7 | 25.8 | Ara-1 | 106.8 | 105.5 | 107.2 | 103.8 |
| 3 | 82.1 | 91.3 | 82.4 | 82.0 | 2 | 73.2 | 79.2 | 73.7 | 81.2 |
| 4 | 43.6 | 43.7 | 44.0 | 43.4 | 3 | 74.8 | 73.8 | 75.3 | 73.5 |
| 5 | 47.7 | 57.2 | 48.1 | 47.8 | 4 | 69.8 | 69.1 | 70.2 | 68.2 |
| 6 | 18.3 | 19.4 | 18.7 | 18.1 | 5 | 67.1 | 65.4 | 67.6 | 64.7 |
| 7 | 32.6 | 35.6 | 33.1 | 33.1 | Glc I-1 |  | 104.9 |  | 105.8 |
| 8 | 40.1 | 40.7 | 40.5 | 39.6 | 2 |  | 76.1 |  | 76.1 |
| 9 | 48.3 | 52.1 | 48.7 | 48.0 | 3 |  | 78.1 |  | 78.2 |
| 10 | 37.1 | 38.2 | 37.5 | 36.8 | 4 |  | 71.1 |  | 71.2 |
| 11 | 24.0 | 22.3 | 24.4 | 23.7 | 5 |  | 78.3 |  | 78.1 |
| 12 | 123.0 | 27.0 | 123.5 | 122.4 | 6 |  | 63.1 |  | 62.4 |
| 13 | 144.3 | 39.5 | 144.7 | 144.7 | 28-O-sugar |  |  |  |  |
| 14 | 42.3 | 42.3 | 42.7 | 42.0 | Glc II-1 | 95.7 | 95.4 | 96.3 |  |
| 15 | 28.4 | 31.0 | 28.8 | 28.5 | 2 | 74.1 | 74.2 | 74.7 |  |
| 16 | 23.4 | 33.0 | 23.9 | 23.5 | 3 | 78.4 | 78.4 | 79.9 |  |
| 17 | 47.1 | 58.1 | 47.5 | 46.6 | 4 | 70.4 | 71.9 | 71.6 |  |
| 18 | 41.8 | 50.7 | 42.3 | 41.8 | 5 | 78.1 | 78.2 | 79.5 |  |
| 19 | 46.3 | 48.5 | 46.7 | 46.3 | 6 | 69.3 | 69.7 | 62.7 |  |
| 20 | 30.9 | 151.9 | 31.3 | 30.8 | Glc III-1 | 105.0 | 104.7 |  |  |
| 21 | 34.1 | 31.7 | 34.5 | 34.1 | 2 | 75.4 | 75.4 |  |  |
| 22 | 32.9 | 37.8 | 33.4 | 32.7 | 3 | 76.6 | 76.9 |  |  |
| 23 | 64.6 | 28.6 | 65.0 | 64.9 | 4 | 78.8 | 79.7 |  |  |
| 24 | 13.7 | 17.0 | 14.2 | 13.3 | 5 | 77.3 | 77.0 |  |  |
| 25 | 16.3 | 16.8 | 16.7 | 15.9 | 6 | 61.4 | 62.1 |  |  |
| 26 | 17.7 | 16.9 | 18.1 | 17.3 | Rha I-1 | 102.9 | 103.1 |  |  |
| 27 | 26.2 | 15.3 | 26.6 | 26.0 | 2 | 72.7 | 72.6 |  |  |
| 28 | 176.7 | 176.4 | 177.0 | 180.1 | 3 | 72.9 | 72.4 |  |  |
| 29 | 33.2 | 110.6 | 33.7 | 33.1 | 4 | 74.0 | 73.9 |  |  |
| 30 | 23.8 | 19.7 | 24.2 | 23.6 | 5 | 71.0 | 70.8 |  |  |
|  |  |  |  |  | 6 | 18.6 | 18.0 |  |  |

**Table S2** ^13^C- NMR (125 MHz) chemical shifts saponins **7**-**9** in pyridine-*d_5_*

| C | **7** | **8** | **9** | C | **7** | **8** | **9** |
| --- | --- | --- | --- | --- | --- | --- | --- |
| 1 | 38.6 | 39.0 | 38.6 | 3-O-sugar |  |  |  |
| 2 | 27.4 | 28.1 | 26.3 | Ara-1 |  |  | 104.9 |
| 3 | 73.9 | 73.7 | 87.8 | 2 |  |  | 81.3 |
| 4 | 47.7 | 39.3 | 40.3 | 3 |  |  | 73.6 |
| 5 | 48.3 | 55.8 | 55.2 | 4 |  |  | 68.4 |
| 6 | 18.4 | 18.8 | 18.3 | 5 |  |  | 65.1 |
| 7 | 32.3 | 33.4 | 33.1 | Glc I-1 |  |  | 106.1 |
| 8 | 39.7 | 40.0 | 40.3 | 2 |  |  | 76.5 |
| 9 | 47.9 | 47.2 | 48.2 | 3 |  |  | 78.3 |
| 10 | 37.0 | 37.3 | 36.6 | 4 |  |  | 71.4 |
| 11 | 23.1 | 23.8 | 23.7 | 5 |  |  | 78.4 |
| 12 | 122.7 | 122.6 | 122.6 | 6 |  |  | 62.5 |
| 13 | 143.9 | 144.4 | 144.8 | 28-O-sugar |  |  |  |
| 14 | 41.9 | 42.0 | 42.4 | Glc II-1 | 95.4 | 95.7 |  |
| 15 | 28.1 | 36.1 | 28.2 | 2 | 73.1 | 73.8 |  |
| 16 | 23.6 | 74.2 | 24.2 | 3 | 78.0 | 77.9 |  |
| 17 | 46.8 | 49.2 | 47.1 | 4 | 70.6 | 70.0 |  |
| 18 | 41.5 | 41.2 | 42.3 | 5 | 77.8 | 78.6 |  |
| 19 | 46.0 | 47.2 | 46.5 | 6 | 68.9 | 69.2 |  |
| 20 | 30.5 | 30.8 | 30.6 | Glc III-1 | 104.6 | 104.8 |  |
| 21 | 33.7 | 35.8 | 34.2 | 2 | 75.1 | 75.2 |  |
| 22 | 32.6 | 32.1 | 32.9 | 3 | 76.3 | 77.1 |  |
| 23 | 67.6 | 28.6 | 28.4 | 4 | 78.5 | 78.2 |  |
| 24 | 12.9 | 16.5 | 16.7 | 5 | 76.9 | 76.4 |  |
| 25 | 15.9 | 15.7 | 15.6 | 6 | 61.0 | 61.2 |  |
| 26 | 17.4 | 17.5 | 17.4 | Rha I-1 | 102.5 | 102.6 |  |
| 27 | 25.8 | 27.1 | 26.3 | 2 | 72.3 | 72.5 |  |
| 28 | 176.3 | 176.0 | 180.2 | 3 | 72.5 | 72.7 |  |
| 29 | 32.9 | 33.1 | 33.4 | 4 | 73.7 | 73.9 |  |
| 30 | 23.5 | 24.5 | 23.7 | 5 | 70.1 | 70.2 |  |
|  |  |  |  | 6 | 18.3 | 18.5 |  |
